# Management of Concomitant Severe Thermal Injury and ST-Elevation Myocardial Infarction

**DOI:** 10.3390/ebj5020015

**Published:** 2024-06-04

**Authors:** Julie Beveridge, Curtis Budden, Abelardo Medina, Kathryne Faccenda, Shawn X. Dodd, Edward Tredget

**Affiliations:** 1 Division of Plastic and Reconstructive Surgery, University of Alberta, Edmonton, AB T6G 2B7, Canada; 2 Department of Anesthesiology & Pain Medicine, University of Alberta, Edmonton, AB T6G 2B7, Canada; faccendak@gmail.com

**Keywords:** thermal injury, burn, myocardial infarction, dual antiplatelet therapy, cardiac disease, acute coronary syndrome, anticoagulation

## Abstract

Acute coronary thrombosis is a known, but rare, contributor to morbidity and mortality in patients with thermal and electrical injuries. The overall incidence of myocardial infarction among burn patients is 1%, with an in-hospital post-infarction mortality of approximately 67%, whereas the overall mortality rate of the general burn patient population is from 1.4% to 18%. As such, early detection and effective peri-operative management are essential to optimize patient outcomes. Here, we report the details of the management of an adult male patient with a 65% total body surface area severe thermal injury, who developed an ST-elevation myocardial infarction (STEMI) in the resuscitation period. The patient was found to have 100% occlusion of his left anterior descending coronary artery, for which prompt coronary artery stent placement with a drug-eluting stent (DES) was performed. Following stent placement, the patient required dual antiplatelet therapy. The ongoing dual antiplatelet therapy required the development of a detailed peri-operative protocol involving pooled platelets, packed red blood cells, desmopressin (DDAVP™) and intraoperative monitoring of the patient’s coagulation parameters with thromboelastography for three staged operative interventions to achieve complete debridement and skin grafting of his burn wounds.

## 1. Introduction

Acute coronary thrombosis is a known, but rare, contributor to morbidity and mortality in patients with thermal and electrical injuries [1,2,3]. Although 13% of thermal injury patients develop some type of cardiac event during admission, the reported incidence of myocardial infarction after thermal injury is only 1% [1,2,3]. Among the 1% of burn patients who sustain a myocardial infarction, however, the post-infarction mortality rates range from 67% to 71% [3,4]. This post-infarction mortality rate is significantly greater than the overall mortality rate of patients with severe thermal injury, which ranges from 1.4% to 18%, as reported in the literature [2,5,6,7]. Despite the alarming mortality rates associated with myocardial infarctions in burn patients, limited evidence exists as to the best management of acute coronary thrombosis in the post-severe burn injury patient. As such, burn patients are often managed in traditional means, which include dual antiplatelet therapy in conjunction with coronary artery stenting. The challenge associated with the traditional management of acute coronary thrombosis is that it complicates the optimal surgical management of burn injuries due to the increased risk of bleeding. This is particularly relevant in the presence of severe burns, as strategies to balance bleeding risk versus cardiac infarct risk during the surgical management of large-area burns are not well delineated in the literature.

The following case report details the management of an adult male patient with a 65% total body surface area (TBSA) severe thermal injury, who developed ST-elevation myocardial infarction (STEMI) in the acute post-burn period.

## 2. Case Report

A 56-year-old male patient sustained a self-inflicted 65% TBSA full-thickness burn to his back and circumferential bilateral arms and deep partial-thickness burns to his anterior chest, neck and posterior thighs. He was immediately transported to the University of Alberta Hospital, a major Canadian burn center, for resuscitation, where emergent intubation and bilateral upper extremity escharotomies were performed. Apart from a history of chronic back pain, he otherwise had no past medical history. Initial resuscitation was guided by the American Burn Association and International Society for Burn Injury recommendations. Fluid resuscitation was first started with 500 mL/hour of Ringer’s lactate in the field. Upon arrival to the Emergency Department, a temperature-sensing Foley Catheter was inserted. The volume of fluid resuscitation was then titrated to the urine output, with a goal of 0.3–0.5 mL/kg/hour. Warming techniques were employed to ensure a core temperature of >36.5 °C. An arterial line was inserted, and the blood pressure goals were >90 mmHg systolic pressure and a mean arterial pressure of >65 mmHg. Initial arterial blood gas analysis revealed a hemoglobin level of 184 g/L and a hematocrit of 0.56 L/L.

Despite aggressive fluid resuscitation, the intermittent use of norepinephrine was required to meet the blood pressure goals. The dosing ranged from 0.01 to 0.3 mcg/kg/minute. In addition to norepinephrine and Ringer’s lactate, albumin infusion was also initiated with (0.5 mL × kg × %TBSA)/24 h of 5% albumin.

The patient remained intubated throughout the resuscitation period. A maximum fraction of inspired oxygen (FiO_2_) of 1.0 and a positive end-expiratory pressure of 12 mmHg were required. The patient required pressure support throughout his resuscitation.

Five hours post-injury, while undergoing resuscitation, the patient was noted to have developed ST-segment elevation on cardiac monitoring. An electrocardiogram was obtained, which revealed evidence of an extensive anterior wall myocardial infarction. As such, he was assessed by a cardiologist, and an urgent echocardiogram was performed, revealing anterior and anteroseptal hypokinesis and apical akinesis with a left ventricular ejection fraction of 50%. As such, the patient underwent emergency cardiac catheterization, through which he was found to have 100% occlusion of his left anterior descending coronary artery, requiring the placement of a drug-eluting stent (DES). The prompt coronary artery placement of a DES resulted in no enduring adverse echocardiogram changes. Beta-blockers were not given until three weeks post-myocardial infarction due to the intermittent need for salbutamol to support ventilation in the context of a known inhalational injury and persistent hypotension. Once the patient was stabilized, bisoprolol was initiated given its cardioprotective role post-myocardial infarction and its ability to reduce catabolism in the context of an increase in the plasma catecholamine levels post-burn [7].

In the burn shock phase of care, the patient had a very tumultuous course in the Intensive Care Unit due to pulmonary failure secondary to the inhalational injury and aggressive fluid resuscitation, with abnormal cardiac function. Three days post-injury, his oxygen requirement decreased from FiO_2_ 1.0 to 0.4 and surgical planning took place. In consultation with a cardiologist, it was determined that the risk of coronary artery stent occlusion with stopping dual antiplatelet therapy (aspirin and clopidogrel) peri-operatively was too high. A thorough and detailed discourse regarding the patient’s prognosis with and without surgery was discussed amongst plastic surgery, anesthesia, hematology, transfusion medicine specialists and the patient’s family. Because of the evolving sepsis and deep thermal wounds, it was considered that the risk of mortality secondary to burn wound sepsis outweighed the risk of surgical intervention.

On post-injury day six, the patient was considered stable enough to proceed with operative intervention. A detailed peri-operative protocol was developed, according to which the patient was transfused with 1 unit of pooled platelets immediately prior to surgery as well as with desmopressin (DDAVP™) 20 mcg intravenously 45 min prior to surgery. Intraoperatively, an additional unit of pooled platelets was transfused, as well as any additional blood products needed. Intra-operative thromboelastography was performed in order to monitor the patient’s coagulation profile in real time. Packed red blood cells (pRBCs) and fresh frozen plasma (FFP) were present in the operating room with the patient before a trial of fascial debridement of one upper extremity under tourniquet with allograft coverage was performed. Hemostasis was maintained after tourniquet release without major difficulty, and the patient remained stable intra-operatively. Therefore, the patient was turned to the prone position, and fascial excision was performed of the entire third-degree burn on his back, followed by split-thickness autograft skin grafting from lower extremity donor sites. Both thighs were insufflated with 4 L of saline with 1:400,000 epinephrine using a cardiac bypass pump equipped with a counter-current heating device, prior to graft harvest.

Hemostasis was achieved by cauterization, and Quick-clot© (Morrisville, NC, USA) was applied to all sites of excision as well as to the donor sites. Intra-operatively, the patient required 11 L of crystalloid in the form of Plasmalyte™, one pool of platelets, 1 unit of FFP, and 2 units of pRBCs. The patient recovered uneventfully in the post-operative period, with no uncontrolled blood loss and minimal transfusion requirements. The next operative intervention was performed on post-injury day ten, in which the patient underwent tracheostomy and dressing change to both upper extremities, with good allograft take. Thereafter, debridement of his neck, chest, abdomen, and shoulders was performed, and frozen allograft utilized for coverage. The remaining burn wounds to both lower extremities received coverage with split-thickness autografts. A similar operative protocol was adhered to, including intra-operative monitoring with TEG and careful hemostasis. The patient remained stable and required one pool of platelets, 1 g of tranexamic acid and 4 units of pRBCs intra-operatively. Post-operatively, the patient developed significant bleeding of the chest and abdominal wounds, requiring bedside cautery, topical epinephrine and topical tranexamic acid before the bleeding was controlled. After this operative procedure, debridement and coverage of the entirety of the burn wounds was achieved.

On post-injury day 23, after re-epithelialization of the patient’s donor sites, the remaining allograft was debrided and replaced with split-thickness autografts using a similar peri-operative and intra-operative strategy. The patient was transferred out of the intensive care portion of the burn unit on post-admission day thirty and discharged to a burn-specific rehabilitation program forty-five days post-injury.

A multi-disciplinary approach allowed this patient’s thermal injury to be successfully debrided and grafted. Strict adherence to the pre-operative administration of platelets and DDAVP™, in addition to intraoperative monitoring of the coagulation parameters and the administration of platelets, as well as a meticulous operative technique, allowed for adequate hemostasis, despite continuation of the dual antiplatelet therapy.

## 3. Discussion

The mechanism underlying coronary thrombosis after thermal injury has yet to be fully elucidated; however, numerous potential abnormalities typical of burn patients may contribute to it. Patients having suffered a severe burn undergo a significant amount of cardiac stress due to both rapid fluid shifts and severe hypovolemia [8,9]. Moreover, this cardiac stress is propagated by the 10- to 20-fold surge of plasma catecholamines—principal mediators of the post-burn hypermetabolic response [8,9]. These high circulating levels of endogenous catecholamines typical of the burn patient may play a role, both directly and indirectly, in myocardial infarction [10]. The release of multiple inflammatory mediators including IL-1 and TNF-alpha from burn wounds places burn victims at risk for myocardial infarction by similar mechanisms to those activated in patients with unstable angina [11]. Such changes in cardiac physiology after severe burn injury may persist for up to two years [12].

In the case where burn patients do suffer a myocardial infarction requiring the placement of a drug-eluting coronary artery stent (DES) [13], the surgical management of their burns is complicated by the requirement of dual antiplatelet therapy. Stent thrombosis is a sudden and potentially catastrophic complication of PCI, which usually manifests as ST-segment elevation myocardial infarction (STEMI), malignant arrhythmias, and/or death [14]. Stent thrombosis is a platelet-mediated process that occurs via platelet activation and aggregation leading to thrombus formation [15]. Moreover, PCI causes endothelial and intimal damage that heals by neointimal formation, usually within 2 to 6 weeks, with bare-metal stent placement [16]. However, with a DES, there is a reduction in platelet aggregation and thrombus formation delaying reendothelialization and neointimal repair, which causes the need for prolonged dual antiplatelet therapy. As such, both the American and the European guidelines recommend the use of dual antiplatelet therapy in patients with a DES for at least 12 months after percutaneous coronary intervention (PCI) [17,18,19,20,21,22,23,24].

The current recommendations by the European Society of Anaesthesiology and Intensive Care regarding urgent surgery following the placement of a DES are that if surgery cannot be avoided or delayed, maintaining dual antiplatelet therapy is of paramount importance [17]. This applies to most surgical procedures, except those in areas where bleeding is in a closed space and might be catastrophic, such as intracranially [21]. For intermediate-risk surgery, such as debridement and skin grafting of burns, the consensus in the literature is that the continuation of dual antiplatelet therapy should be performed with consultation of the cardiology and surgical teams, as in this case [22,23]. Given the high risk of peri-operative hemorrhage in this case, DDAVP™ and platelet administration were used pre-operatively to mitigate the risk. At least 50% of the platelets must be functional in order to obtain adequate hemostasis. Due to the irreversibility of antiplatelet agents, fresh platelets are the most efficient way to re-establish normal coagulation. The continuation of dual antiplatelet therapy post-operatively ensured that the transfused platelets were inhibited before the maximal prothrombotic phase after surgery was reached (typically 2–4 days post-surgery) [24].

Desmopressin’s (DDAVP™) primary mechanism of action is to increase the circulating levels of factor VIII and von Willebrand factor (vWf), leading to secondary improvements in platelet adhesion to endothelial defects [25,26]. Ranucci et al. reported a single case on the use of DDAVP™ to reverse the effects of clopidogrel in a patient undergoing emergent carotid endarterectomy [27]. They described the abnormal results of a platelet function assay consistent with the clopidogrel therapy. These abnormalities improved following the administration of a single dose of 0.3 mcg/kg of DDAVP™ but did not reverse completely. Floradal et al. investigated the effect of DDAVP™ on bleeding time in patients undergoing elective open cholecystectomy [28]. Six of the patients taking aspirin pre-operatively were randomized to receive two doses of DDAVP™ (0.3 mcg/kg/dose over 30 min) at induction of anesthesia and then 6 h later. DDAVP™ administration improved the bleeding time compared to placebo. With the increasing use of antiplatelet agents such as clopidogrel and aspirin, the potential role of DDAVP™ in the acute setting to control bleeding appears promising; however, there is a paucity of randomized controlled trials evaluating its safety and efficacy in the burn population [29]. Despite limited evidence for the effectiveness of DDAVP™ in the reversal of dual antiplatelet therapy, it was felt that the potential benefit of the use of DDAVP™ in this case was favorable, given the medications’ low risk profile.

The intra-operative monitoring of the patient’s coagulation parameters with thromboelastography (TEG) was imperative to the successful operative management of this patient. Thromboelastography is a viscoelastic hemostatic assay that measures the global viscoelastic properties of whole blood clot formation under low shear stress, demonstrating the interaction of platelets with the coagulation cascade (aggregation, clot strengthening, fibrin cross-linking and fibrinolysis) [30]. TEG measures not only clotting factor functions similar to INR and PTT, but also fibrinolysis and platelet function. Platelet-mapping TEG aims to determine to what degree platelet function may be inhibited due to pharmacologic inhibition of either the arachidonic acid or the adenosine diphosphate pathway. This function was of particular use in this case, as it enabled our anesthesia team to determine if and when more platelets needed to be given in order to achieve hemostasis. Schaden et al. demonstrated that a bleeding management algorithm based on thromboelastometry is efficacious in reducing the need for allogenic blood product requirements in burn patients undergoing surgical excision when compared to clinical discretion [31]. Shore-Lesserson et al. previously demonstrated this in the non-burn population [32]. TEG is commonly employed for monitoring the coagulation parameters of trauma patients undergoing surgery [33] and, for these reasons, should be given serious consideration in moderate-to-high-risk surgical interventions, particularly with patients requiring dual antiplatelet therapy.

## 4. Conclusions

This case report describes the management of an uncommon, but ultimately life-threatening, complication of severe burn injury secondary to acute STEMI. Treatment of this patient’s STEMI with a drug-eluting stent required the subsequent continuation of dual antiplatelet therapy, as the risk of discontinuing the antiplatelet therapy prior to skin graft surgery was considered prohibitive. However, a multidisciplinary operative strategy, including strict adherence to pre-operative blood product administration and a staged operative approach with meticulous hemostasis, led to a successful outcome for this severely burned patient.

## Data Availability

Data are contained within the article.
